# Mosquito-Based Detection of Endogenous Jaagsiekte Sheep Retrovirus in Senegal: Expanding the Scope of Xenosurveillance

**DOI:** 10.21203/rs.3.rs-5951454/v1

**Published:** 2025-04-25

**Authors:** Marie Henriette Dior Ndione, El Hadji Ndiaye, Madeleine Dieng, Babacar Diouf, Safietou Sankhé, Diawo Diallo, Mouhamed Kane, Ndeye Marie Sene, Maimouna Mbanne, Faty Amadou Sy, Seynabou Mbaye Ba Souna Diop, Serge Freddy Moukaha Doukanda, Amadou Alpha Sall, Ousmane Faye, Ndongo Dia, Scott C Weaver, Oumar Faye, Mawlouth Diallo, Gamou Fall, Alioune Gaye, Moussa Moise Diagne

**Affiliations:** Institut Pasteur de Dakar; Institut Pasteur de Dakar; Institut Pasteur de Dakar; Institut Pasteur de Dakar; Institut Pasteur de Dakar; Institut Pasteur de Dakar; Institut Pasteur de Dakar; Institut Pasteur de Dakar; Institut Pasteur de Dakar; Institut Pasteur de Dakar; Institut Pasteur de Dakar; Institut Pasteur de Dakar; Institut Pasteur de Dakar; Institut Pasteur de Dakar; Institut Pasteur de Dakar; University of Texas Medical Branch; Institut Pasteur de Dakar; Institut Pasteur de Dakar; Institut Pasteur de Dakar; Institut Pasteur de Dakar; Institut Pasteur de Dakar

**Keywords:** Jaagsiekte sheep Retrovirus, xenosurveillance, mosquitoes, Senegal, metagenomics, virus–host–vector interactions

## Abstract

**Background:**

Mosquitoes are well-known vectors for arthropod-borne viruses, yet their role as passive carriers of non-arthropod-borne viruses remains underexplored. Xenosurveillance, a method that utilizes blood-feeding arthropods to sample host and pathogen genetic material, has emerged as a valuable tool in viral ecology. In this study, we report the first identification of Jaagsiekte Sheep Retrovirus (JSRV)-related sequences in blood-fed mosquitoes collected in Senegal. JSRV, a betaretrovirus responsible for ovine pulmonary adenocarcinoma, is typically found in sheep, but its genetic trace in mosquitoes offers a novel perspective on host–vector contact and surveillance. Our study aimed to investigate whether mosquitoes can serve as sentinels for detecting both pathogens and host-derived markers in complex ecosystems.

**Methods:**

Mosquitoes were collected between 2016 and 2019 from three ecologically significant regions in Senegal (Louga, Barkedji, and Kedougou). Blood-fed mosquitoes were pooled and subjected to RNA extraction and metagenomic sequencing using Illumina NextSeq550. Sequencing data were analyzed with CZ-ID and BLAST for viral identification. RT-qPCR assays were designed to validate the presence of JSRV-related sequences, targeting conserved regions of the envelope gene and 3’ untranslated region. Phylogenetic analysis was conducted using MAFFT and IQ-TREE to compare the detected sequence with global exogenous and endogenous JSRV references.

**Results:**

A diverse array of viruses across mosquito species, including both arboviruses and non-arthropod-borne viruses. A JSRV-related sequence was detected in a single blood-fed mosquito pool collected in Barkedji (2019). The RT-qPCR assay confirmed JSRV presence, validating the sequencing results. Phylogenetic analysis revealed strong similarity to known endogenous JSRV (enJSRV) sequences integrated in the sheep genome, indicating that the detected material likely originated from host DNA ingested during blood feeding.

**Discussion:**

This study presents the first report of endogenous retroviral sequences detected in mosquitoes, alongside the identification of actively circulating viruses, highlighting the broader potential of mosquitoes as environmental sentinels. While mosquitoes are not biological vectors for JSRV, their ability to capture both host-derived retroviral material and pathogenic viral genomes through bloodmeals reinforces the value of xenosurveillance for monitoring livestock–vector–environment interactions. These findings contribute to broader efforts in integrated disease surveillance and underscore the utility of combining metagenomics with molecular diagnostics to detect diverse viral signals in high-risk ecological settings.

## Background

The discovery of viruses transmitted by arthropod vectors has transformed our understanding of viral ecology and infectious disease dynamics [1]. Mosquitoes, in particular, play a critical role in transmitting a wide range of pathogens that affect both animal and human populations [2]. This fact has prompted extensive research into the complex interactions between vectors, viruses, and hosts, with a special focus on zoonotic spillover events where pathogens cross species barriers [3]. Advances in next-generation sequencing (NGS) technologies have further accelerated pathogen discovery, especially in the context of vector-borne diseases. NGS enables high-throughput, unbiased sequencing, allowing for the detection of both known and novel viruses in diverse ecological settings [4].

Xenosurveillance, or the sampling of pathogen genetic material from the blood meals of arthropod vectors, has proven effective for detecting viruses circulating in wildlife and domestic animals [5, 6]. When combined with metagenomic sequencing, xenosurveillance offers comprehensive insights into viral diversity in specific environments. This approach not only aids in the discovery of new pathogens but also helps clarify viral transmission ecology, identifying potential vectors and reservoirs for zoonotic diseases [7].

Jaagsiekte sheep retrovirus (JSRV) is a betaretrovirus known to cause ovine pulmonary adenocarcinoma (OPA), a contagious lung cancer in sheep [8]. Transmission occurs primarily via aerosolized droplets, and the virus has been considered tightly restricted to sheep and closely related ruminants. Two forms of JSRV exist: the exogenous, infectious virus (exJSRV), and multiple endogenous variants (enJSRVs), which are stably integrated into the host genome. These endogenous forms share high sequence similarity with exJSRV and retain some functional elements, raising interest in their ecological relevance and evolutionary relationship. Traditionally, arthropods have not been implicated in JSRV transmission, and no vector-borne involvement has been demonstrated.

In this study, alongside other arthropod-borne and non-arthropod-borne viruses, we report the first detection of JSRV-related genetic material in blood-fed mosquitoes collected in southeastern Senegal, identified through a vector-enabled metagenomic approach. Phylogenetic analysis revealed that the sequence is most closely related to enJSRV. While this finding does not indicate active viral replication or vector-borne transmission, it provides indirect evidence of mosquito contact with local ruminant hosts, particularly sheep. This supports the growing utility of xenosurveillance for capturing host-derived genetic signatures, in addition to circulating pathogens. Moreover, it aligns with emerging ecological observations, such as the recent detection of JSRV RNA in surface water from Tanzanian waterholes during dry seasons [9], which together raise important questions about non-traditional interfaces of viral exposure and environmental persistence in pastoral ecosystems.

By capturing host-derived retroviral sequences through mosquito metagenomics, our work underscores the broader potential of xenosurveillance to advance understanding of host–vector–environment interactions and to support integrated pathogen and exposure monitoring systems at the intersection of animal and public health.

## Methods

### Study sites

Blood-fed mosquitoes were collected between 2016 and 2019 from three ecologically significant regions in Senegal, Louga in the northwest, Barkedji in the central Ferlo region, and Kedougou in the southeast, each chosen for their relevance to vector-borne disease dynamics. Louga serves as a transitional zone between agriculture and pastoralism, where fluctuating rainfall and rising temperatures impact livestock and crop production, fostering mosquito-host interactions [10]. Barkedji, centrally located, features pastoral corridors and wildlife reserves, providing a critical interface for zoonotic spillovers [11]. Pastoral activities in the region are marked by seasonal livestock movements, with sheep and goats forming the backbone of herds due to their resilience to water scarcity. Kedougou lies within a tropical forest zone near the Guinean border, where the humid climate supports high mosquito activity, making it a hotspot for pathogen circulation [12, 13]. Although pastoralism is less dominant in this area compared to the Ferlo or Louga regions, Kedougou has become an important destination for herders from northern Senegal due to its abundant pasture and water resources, especially during dry seasons. Herders are drawn to Kedougou for economic opportunities, including livestock markets where animals command attractive prices, enabling them to diversify their income sources [14].

### Mosquito collection and sample selection

This study analyzed archived mosquito pools collected biweekly between 2016 and 2019 as part of the national arbovirus surveillance program coordinated by IPD. Mosquitoes were collected using a variety of trapping methods described by Ndiaye et al., including CDC light traps baited with CO_2_, placed at both ground and canopy levels [15]. Traps were deployed near livestock enclosures, village edges, and natural water sources in ecologically diverse regions such as Barkedji, Louga, and Kedougou.

Following morphological identification on the field, mosquito pools were archived at the Centre de Recherche sur les Arbovirus (CRORA) biobank [16]. For this study, a subset of pools was pre-selected based on their ability to induce cytopathic effect (CPE) upon inoculation in mosquito and mammal cell culture but had tested negative by indirect immunofluorescence assay (IFA) for common arboviruses [17]. This selection strategy prioritized samples likely to contain uncharacterized or non-target viral agents.

Whole mosquito specimens from the selected pools were homogenized, and total nucleic acids were extracted for metagenomic sequencing to identify both host- and pathogen-derived viral material.

### RNA Extraction and Metagenomic Sequencing

RNA extraction was performed using the MagMax Viral/Pathogen Nucleic Acid Isolation Kit and the KingFisher Flex Purification System (Thermo Fisher), following the manufacturer’s recommendations. Sequencing was conducted on the extracted RNA using an Illumina-based unbiased approach, as described previously [18]. Briefly, host ribosomal RNA was depleted using specific rRNA-targeting probes [19] and RNase H, and the remaining RNA served as the template for first-strand cDNA synthesis with the SuperScript IV Reverse Transcriptase kit. Double-stranded cDNA was then generated using Klenow fragment DNA polymerase. Libraries were prepared with the Nextera XT DNA Library Preparation kit (Illumina), and sequencing was carried out on the NextSeq 550 system.

The resulting sequences were analyzed using the CZ-ID metagenomics platform (http://czid.org, accessed on 15 March 2023) for viral identification [20]. Default pipeline parameters were used, incorporating quality control steps such as adapter trimming, low-complexity read filtering, and the removal of host-derived reads (sheep, goat, human) as well as common laboratory contaminants. Sequence alignment was performed against curated nucleotide (NT) and protein (NR) databases using Bowtie2 and RAPSearch2, respectively. Reads were assembled into contigs with SPAdes, and taxonomic classification was based on nucleotide and translated alignments, applying identity thresholds of approximately 80–85% for NT and 90–95% for NR, with e-value cutoffs set at ≤ 1e-10.

Additionally, the assembled sequences were subjected to BLAST analysis (Basic Local Alignment Search Tool) against the NCBI nucleotide database to confirm viral identity and assess sequence similarity with known viral genomes, facilitating more precise taxonomic classification and strain identification.

### Molecular Assay Development

To confirm the presence of JSRV detected through NGS, we developed two TaqMan-based reverse transcription quantitative PCR (RT-qPCR) assay were developed. These assays utilized primers and a fluorescently labeled probe designed with Primer3 [21], specifically targeting regions of the envelope gene and the 3’ untranslated region (3’UTR) based on our assembled JSRV sequences. The designed oligonucleotide sets were rigorously evaluated in silico for specificity using BLAST analysis, ensuring that they would exclusively bind to the target JSRV sequences without cross-reactivity with other viral or host genomes. In addition to the in-house RT-qPCR, we employed the hemi-nested U3hn PCR, a previously described method targeting the U3 region of the viral long terminal region (LTR), and known for its high sensitivity in field studies [22]. This assay was used to complement and validate the results obtained with our newly developed RT-qPCR assay. All assays were performed on RNA extracted from mosquito pools, providing a robust confirmation framework for the detection of JSRV-related sequences.

### Jaagsiekte virus Phylogenetic Analysis

The consensus data generated during this study were aligned with a representative dataset of available sequences using the multiple sequence alignment tool MAFFT (version 7) [23] with default parameters. This alignment allowed for a comprehensive comparison of the newly detected sequence with known viral genomes, providing a robust framework to establish their evolutionary relationships. The aligned sequences were then used to construct a maximum likelihood (ML) phylogenetic tree, employing IQ-TREE [24], a widely recognized tool for phylogenetic inference. The best-fit substitution model for the analysis was selected using ModelFinder [25], ensuring an accurate representation of the evolutionary processes underlying the data. The phylogenetic tree was visualized using FigTree (version 1.4.4) (http://tree.bio.ed.ac.uk/software/figtree/, accessed on 17 March 2023), a flexible software for exploring and presenting phylogenetic results.

## Results

### Viral Diversity in Mosquito Populations, Co-detection Patterns and Regional Variation

A total of 78 mosquito pools collected between 2016 and 2019 were included in the metagenomic analysis. Although our primary focus was on blood-fed female mosquitoes, some pools also included non-blood-fed females and a few males due to the retrospective nature of the archived material. These pools were derived from diverse mosquito species and sites across Senegal.

Our metagenomic sequencing identified a wide array of viruses across various mosquito species collected from different regions of Senegal between 2016 and 2019 (Table 1). These included both arboviruses and non-arboviruses, demonstrating the remarkable diversity of viral communities harbored by mosquitoes. Notable detections were observed in species such as *Aedes aegypti*, *Aedes vexans*, *Culex quinquefasciatus*, and *Aedes furcifer*, each playing a distinct role in the viral ecosystem. The identified viruses spanned multiple viral species, with significant detections of Pestivirus A, Dezidougou virus, and Culex iflavivirus, reflecting a broad spectrum of viral types circulating in the environment. Co-detections were frequently observed across several mosquito species, suggesting complex viral interactions within vector populations. While multiple viruses were identified in some pooled samples, we refer to these as instances of co-detection rather than confirmed co-infections. Given that each pool may contain multiple mosquitoes, the detected viral sequences could originate from different individuals. As such, these findings reflect aggregate viral presence within the pool and do not indicate concurrent infection of a single mosquito.

The metrics associated with viral identification are provided in supplementary table 1.

For example, Pestivirus A, known as bovine viral diarrhea virus type 1 infecting various vertebrates including cattle, sheep, goats, pigs and wild ruminants was widely detected in mosquitoes from multiple regions, including Kedougou and Barkedji, particularly in species like *Aedes unilineatus* and *Aedes furcifer*. Dezidougou virus, another frequently identified virus, was found alongside Aedes vexans iflavivirus in *Aedes vexans* pools from Barkedji. In *Culex quinquefasciatus* from Louga, co-detections were observed, with Culex iflavivirus and Pestivirus H appearing in the same pools as Hubei partiti-like virus and Avian Leukosis virus, highlighting the complexity of viral ecosystems in the region.

These findings underscore the extensive viral diversity present within mosquito populations, with marked regional variation observed across the study sites. Distinct viral profiles were found in Barkedji (51 pools, 1,085 mosquitoes), Louga (19 pools, 192 mosquitoes), and Kedougou (8 pools, 70 mosquitoes), likely influenced by local ecological factors. Barkedji and Louga each harbored 13 unique viral species, while Kedougou, a tropical forest site, had only 3. This may reflect our sampling strategy, which focused on pools that induced cytopathic effects in mosquito or mammalian cell cultures but tested negative for common arboviruses, enhancing the detection of uncharacterized or non-target viruses. Such variation points to the critical role of regional climate, host availability, and habitat conditions in shaping mosquito-associated viral communities.

Notably, co-detections within the same mosquito pools were more common in samples from Louga and Barkedji, suggesting that these regions may be hotspots for viral transmission and co-circulation. While we cannot infer true co-infections at the individual mosquito level due to the use of pooled samples, these patterns may reflect overlapping ecological niches or shared host exposure, facilitating the simultaneous presence of multiple viral species in the same sampling units.

### Detection of Jaagsiekte Sheep Retrovirus

Among the various viruses identified, our analysis revealed the first detection of JSRV in mosquitoes. From a blood-fed mosquito pool collected in Barkedji in 2019, we recovered a partial genome sequence (about 600 base pairs in length) encompassing the terminal region of the *Env* gene and 3’UTR of JSRV. Based on these findings, we developed two sets of molecular assays to confirm the taxonomic identity of JSRV (Table 2).

To further support these results, we also employed the previously validated U3hn PCR to complement our in-house assays. The U3hn PCR served as an independent molecular confirmation of JSRV presence, particularly useful in low-viral-load samples.

RT-qPCR and U3hn PCR were both performed on the mosquito pool in which JSRV was initially detected, along with all nine additionally blood-fed pools with no prior evidence of infection. The assays confirmed JSRV presence in the original mosquito pool (Supplementary table 2), though follow-up sequencing did not yield additional coverage of the viral genome.

### Phylogenetic Analysis of Jaagsiekte Sheep Retrovirus

To further characterize the JSRV-related sequence detected in mosquitoes, we performed a phylogenetic analysis using a multiple sequence alignment covering a region of the partial Env gene and the LTR. This alignment included reference betaretroviruses, with particular emphasis on both exJSRV and endogenous (enJSRV) JSRV sequences. A ML phylogenetic tree was then constructed to determine the evolutionary placement of the mosquito-derived sequence.

**Figure 1:**
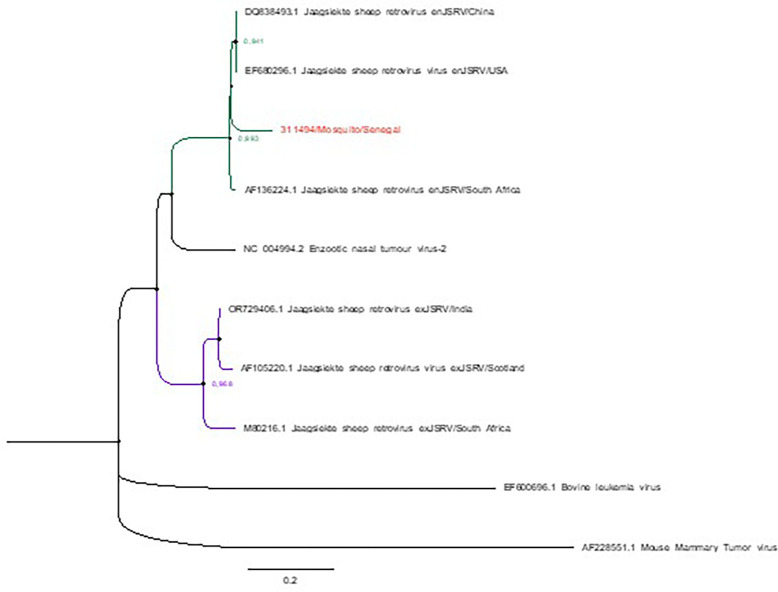
**Phylogenetic analysis of Jaagsiekte Sheep Retrovirus (JSRV)**-related sequence detected **in mosquitoes from Senegal.** Maximum likelihood tree constructed from partial Env–LTR sequences, including the mosquito-derived sequence and representative **exogenous (exJSRV, shown with purple branches), endogenous (enJSRV, shown with green branches), and outgroup** betaretroviruses (shown with black branches). The Senegalese JSRV sequence clusters closely with known **endogenous JSRVs**, supporting its origin from host genomic material rather than an exogenous infectious strain. Bootstrap values are displayed for key nodes to reflect the robustness of the analysis.

The resulting tree (Fig. 1) revealed that the sequence clusters closely with enJSRV strains integrated in the sheep genome, rather than with known exogenous, infectious variants (exJSRV). High bootstrap support (> 90%) on key branches further confirms the robustness of these evolutionary relationships.

## Discussion

This study provides significant insights into the viral ecosystems within mosquito populations in Senegal, identifying a diverse array of viruses, including Pestivirus A, Dezidougou virus, and Culex iflavivirus. The extensive detection of these viruses across various mosquito species, underscores the complexity of viral interactions within vector populations. In the context of xenosurveillance, platforms like CZ-ID provide a standardized approach for detecting viral sequences in blood-fed mosquitoes. However, reliance on default parameters may reduce sensitivity to highly divergent or novel viruses, particularly in ecologically rich settings, potentially leading to an underestimation of viral diversity, a limitation we acknowledge in our interpretation. These findings highlight the critical role that mosquitoes play, not only as vectors of well-established arboviruses but also as reservoirs or passive carriers of non-arboviral pathogens [6]. This emphasizes the need for broader surveillance efforts to understand the contributions of mosquito populations to viral ecology and pathogen transmission.

The detection of JSRV-related genetic material in mosquitoes through unbiased metagenomic sequencing adds a novel dimension to vector-based surveillance. Phylogenetic analysis indicates that the detected sequence is most closely related to enJSRV, non-infectious retroviral elements integrated into the sheep genome. While this does not suggest active viral replication or vector-borne transmission, it provides indirect molecular evidence of mosquito feeding on local ruminant hosts, particularly plausible in Barkedji, a pastoral region characterized by seasonal livestock movements and a high density of sheep and goats. This finding highlights the potential of mosquitoes to act as biological samplers, capturing host-derived viral sequences through bloodmeals, and supports the application of xenosurveillance for ecological monitoring of livestock exposure.

The detection of JSRV-related sequences in mosquitoes also aligns with prior findings from Nigeria and Cameroon, where JSRV-related sequences were identified in human samples [26]. While the biological relevance of those findings remains uncertain, they collectively raise important questions about the distribution and detectability of endogenous retroviral elements across species and environments [27]. In our study, the identification of enJSRV-like sequences in mosquito bloodmeals, alongside the detection of other viruses such as Pestivirus A, Dezidougou virus, and Culex iflavivirus, highlights the value of combining xenosurveillance and metagenomic tools to monitor both actively circulating viruses and genomic material derived from host organisms. This approach provides a window into local livestock exposure and virus–host–vector interactions without the need for direct sampling of animals. Further investigation is warranted to better understand the ecological roles of retroviruses like enJSRV and their incidental detection in non-host organisms such as arthropod vectors [28]. Previous studies in Senegal have reported the presence of ovine betaretroviruses, such as ENTV, directly in infected tissues [29]. By expanding this lens to vector-based environmental surveillance, our findings underscore the potential of mosquitoes to act as passive carriers and ecological sentinels, capturing signals of both active infections and host genomic signatures that may otherwise go undetected.

The utility of xenosurveillance as a tool for pathogen detection is further exemplified in this study. By analyzing viral genetic material obtained from blood-feeding mosquitoes, we were able to identify viruses circulating within local wildlife and domestic animal populations without the need for direct mammalian sampling. This method offers a less invasive and more practical approach to surveillance, particularly in remote or logistically challenging regions. Xenosurveillance not only expands our understanding of viral geographic distribution and host interactions but also enhances the early detection of emerging zoonotic threats [30]. This approach is especially relevant in areas with high biodiversity and frequent interactions between wildlife, domestic animals, and humans, such as Senegal.

Beyond its contributions to pathogen discovery, this study emphasizes the critical role of molecular diagnostics in enhancing viral surveillance. The development of specific molecular assays targeting JSRV-related sequences, informed by NGS data, enabled us to confirm the presence of endogenous retroviral material in mosquito samples. This integration of high-throughput sequencing with targeted molecular tools exemplifies a robust approach not only for pathogen detection but also for identifying host-derived genetic signatures within vector populations. It also underscores the importance of adapting diagnostic approaches to capture both arboviral and non-arboviral elements, thereby expanding the scope of mosquito-based surveillance beyond classical vector-borne disease paradigms [31]. The implications of this study extend to public health preparedness, as the early detection of emerging viral threats can inform targeted interventions and outbreak prevention strategies.

Ultimately, this work highlights the transformative potential of integrating xenosurveillance with next-generation sequencing and molecular diagnostics to enhance our understanding of virus-vector-host interactions. By detecting host-derived endogenous retroviral sequences in mosquito bloodmeals, xenosurveillance can provide insight into both pathogen presence and host exposure, capturing ecological contact patterns that extend beyond classical infection-based surveillance. This approach offers a scalable and minimally invasive tool for monitoring livestock health and supporting early-warning systems at the intersection of animal and public health, particularly in pastoral settings where direct sampling remains a challenge. As global health systems move toward integrated One Health frameworks, mosquito-based xenosurveillance offers a unique opportunity to strengthen disease surveillance across species and environments.

## Figures and Tables

**Table 1:** Mosquito Species and Viruses Identified Across Collection Sites (2016–2019)

Mosquito species	Collection locality	Sex	Date of Collection (Year-Month)	Identified Viruses	Number of pools	Number of individuals	Feeding Status
*Aedes aegypti*	Louga	Female	2017–10	-	2	2	Fed-blood
		Male		Aedes Albopictus Densovirus / Dezidougou virus	1	1	Unfed
			2017–11	Dezidougou virus	1	2	Unfed
				Pestivirus A	1	1	Unfed
	Kedougou	Female	2019–10	Pestivirus A	1	2	Unfed
				Pestivirus A / Taesano Aedes Virus	1	2	Fed-blood
				Taesano Aedes Virus	1	4	Unfed
*Aedes centropunctatus*	Kedougou	Female	2019–10	Pestivirus A	1	1	Unfed
*Aedes dalzieli*	Barkedji	Female	2017–08	-	1	5	Unfed
			2019–09	-	1	1	Unfed
	Kedougou		2019–10	Pestivirus A	2	58	Unfed
*Aedes furcifer*	Kedougou	Female	2019–10	Pestivirus A /Mosquito Densovirus BR/07	1	2	Fed-blood
*Aedes ochraceus*	Barkedji	Female	2017–08	-	1	5	Unfed
*Aedes sudanensis*	Barkedji	Female	2017–08	-	1	1	Unfed
			2019–09	Dezidougou Virus / Daezongdong Virus	1	1	Unfed
*Aedes taylori*	Kedougou	Female	2019–10	-	1	1	Fed-blood
**Aedes unilineatus**	**Barkedji**	**Female**	**2019–09**	**Jaagsiekte Sheep Retrovirus** / Dezidougou Virus	**1**	**3**	**Fed-blood**
				Pestivirus A	1	2	Fed-blood
*Aedes vexans*	Barkedji	Female	2016–09	-	6	213	Unfed
			2017–08	-	4	114	Unfed
				Dezidougou virus / Aedes vexans iflavivirus	1	40	Unfed
			2019–08	-	1	50	Unfed
				Dezidougou Virus	4	200	Unfed
			2019–09	-	1	1	Unfed
				Aedes Vexans iflavivirus	1	3	Unfed
				Aedes Vexans iflavivirus / Dezidougou Virus	1	6	Unfed
				Dezidougou Virus	3	71	Fed-blood
*Anopheles pharaonsis*	Barkedji	Female	2017–08	-	1	3	Unfed
		Male		-	1	1	Unfed
*Culex quinquefasciatus*	Louga	Female	2017–10	Culex iflavivirus / Pestivirus H	1	5	Fed-blood
				Hubei partiti-like Virus	1	1	Unfed
				Hubei partiti-like Virus / Avian Leukosis Virus / Yongsan iflavivirus/Culex iflavivirus	1	2	Fed-blood
			2017–11	-	4	66	Fed-blood
				Culex iflavivirus / Avian leukosis Virus	1	39	Fed-blood
		Male	2017–11	-	1	35	Unfed
				Aedes Albopictus Densovirus	1	5	Unfed
				Culex flavivirus /Culex Densovirus/ Mosquito Densovirus BR/07	1	16	Unfed
				Pestivirus H / Hubei mosquito virus / Culex iflavivirus	1	15	Unfed
*Culex tritaeniorhyncus*	Louga	Female	2017–11	-	2	2	Unfed
*Culex antennatus*	Barkedji	Female	2017–08	-	1	1	Unfed
*Culex ethiopicus*	Barkedji	Female	2016–09	-	1	11	-
			2019–09	-	1	25	-
				Pestivirus A / Dezidougou Virus	1	37	-
*Culex neavei*	Barkedji	Female	2019–10	Barkedji Virus / Bagaza Virus / Sindbis Virus / West-Nile Virus	1	50	
				Barkedji Virus / Bagaza Virus / Usutu Virus	1	50	-
*Culex perfuscus*	Barkedji	Female	2016–09	-	1	4	-
*Culex poicilipes*	Barkedji	Female	2017–08	-	2	3	-
				-	1	6	-
			2019–09	-	1	33	-
				Dezidougou Virus / Aedes vexans iflavivirus	1	50	-
				Dezidougou Virus / Daezongdong Virus	1	50	-
				Pestivirus A	2	37	Fed-blood
*Culex quinquefasciatus*	Barkedji	Female	2019–09	Aedes Pseudocutellaris Reovirus / Pestivirus A	1	1	Fed-blood
				Dezidougou Virus	1	1	-
				Pestivirus A	1	1	Fed-blood
*Culex tigripes*	Barkedji	Female	2017–08	-	1	1	-
*Phlebotom sp*	Barkedji	Female	2019–10	-	1	4	-

**Table 2:** Jaagsiekte sheep retrovirus-targeted RT-qPCR assays designed during the study.

RT-PCR set 1	Enveloppe gene	JaagsiekteV_Left primer_1	CGGTTCTGACTGTTGTGCTT
		JaagsiekteV_Right primer_1	CGCAGCTCCCCTCTCTTTAT
		JaagsiekteV_Probe_1	FAM - AACATGTTGCAACACCGACA - BBQ
RT-PCR set 2	3’ untranslated region	JaagsiekteV_Left primer_2	CCTAAGCTCCCTGTCCCG
		JaagsiekteV_Right primer_2	GCCTTCCTTTATTGTGCTGC
		JaagsiekteV_Probe_2	FAM - TGTGAATGTCAGAAGTCACGT - BBQ

## Data Availability

All data generated or analyzed in this study are provided within the article and its supplementary materials, while the genomic data are publicly accessible in the NCBI repository.
